# ISCEV extended protocol for VEP methods of estimation of visual acuity

**DOI:** 10.1007/s10633-020-09780-1

**Published:** 2020-07-16

**Authors:** Ruth Hamilton, Michael Bach, Sven P. Heinrich, Michael B. Hoffmann, J. Vernon Odom, Daphne L. McCulloch, Dorothy A. Thompson

**Affiliations:** 1 Department of Clinical Physics and Bioengineering, Royal Hospital for Children, NHS Greater Glasgow and Clyde, Glasgow, UK; 2 College of Medical, Veterinary and Life Sciences, University of Glasgow, Glasgow, UK; 3 Eye Center, Medical Center – University of Freiburg, Faculty of Medicine, University of Freiburg, Freiburg, Germany; 4 Department of Ophthalmology, Otto-von-Guericke University, Magdeburg, Germany; 5 Center for Behavioral Brain Sciences, Magdeburg, Germany; 6 Departments of Ophthalmology and Neuroscience, School of Medicine, West Virginia University, Morgantown, WV USA; 7 School of Optometry and Vision Science, University of Waterloo, Waterloo, ON Canada; 8 The Department of Clinical and Academic Ophthalmology, Great Ormond Street Hospital for Children, London, UK; 9 University College London Great Ormond Street Institute of Child Health, London, UK

**Keywords:** Clinical standards, Visual evoked potential (VEP), VEP spatial frequency (SF) limit, Visual acuity, Sweep VEP, Step VEP, International Society for Clinical Electrophysiology of Vision (ISCEV), Extended protocol

## Abstract

The International Society for Clinical Electrophysiology of Vision (ISCEV) standard for visual evoked potentials (VEPs) describes a minimum procedure for clinical VEP testing and encourages more extensive testing. This ISCEV extended protocol is an extension to the VEP standard. It describes procedures for recording multiple VEPs to a range of sizes of pattern stimuli to establish the VEP spatial frequency limit (threshold) and for relating this limit to visual acuity.

## Introduction

The International Society for Clinical Electrophysiology of Vision (ISCEV) standard for visual evoked potentials (VEPs) describes a minimum set of tests but encourages the use of additional VEP protocols for clinical testing [1]. This extended protocol describes the VEP spatial frequency (SF) limit, a specialised procedure which is well established and broadly accepted by experts in the field. The protocol was prepared by the authors in accordance with ISCEV procedures (www.iscev.org/standards) and was approved by the ISCEV Board of Directors on May 24, 2020, following a 2-month period of open consultation with the ISCEV membership. The authors have also undertaken a systematic review of VEPs used for acuity estimation to inform this extended protocol, to provide a contemporary review of the extensive literature and to examine how associations between VEP SF limit and visual acuity vary with maturation and with clinical condition [2].

## Scope and applications

VEPs are evoked in the visual cortex and are obtained by processing electroencephalographic (EEG) signals from overlying scalp electrodes. An intact and functioning visual pathway between the macula and the cortex for a specific stimulus can be inferred from the presence of a normal VEP to that stimulus [1]. Stimuli can be configured to measure a VEP SF limit as an estimate of visual acuity: such techniques have been employed for over 40 years [3, 4]. A VEP SF limit can be a fully objective measure which requires less cognitive function or cooperation than behavioural tests of visual acuity. Thus, VEP SF limits are stand-alone measures of visual function which complement behavioural and structural measures. VEP SF limits and visual acuity are not measurements of the same entity due to differences in stimuli, retinal area, fixation duration, level of the visual system assessed and means of defining a threshold. Despite these differences, agreement between VEP SF limits and behavioural measures of acuity can be sufficiently consistent to make VEPs useful for clinical estimation of acuity when behavioural testing is not possible or reliable. An empirical calibration factor or offset (see Response evaluation, part (c)) is usually required to estimate behavioural visual acuity from VEP SF limits: such factors depend on the specific VEP SF limit method, the acuity test, the subject’s age, the type of visual dysfunction and, to a lesser extent, the subject’s acuity. This empirically determined calibration factor is required to infer visual acuity from a VEP SF limit: for example, it is incorrect to assume that a VEP SF limit of 30 cycles per degree (cpd) is equivalent to a visual acuity of 0.0 logMAR, i.e. 1.0 (decimal), 6/6 or 20/20 (Snellen) as this relationship often fails to hold for VEP SF limits.

We have adopted a terminology convention for thresholds, acuity and related measures which uses “good”, “better”, “poor” or “poorer” in preference to “high”, higher”, “low” or “lower” since some units such as the logMAR scale are such that lower numerical values denote better performance. Pattern element sizes are described as “coarse” or “fine” in preference to “high” or “low” since SF units such as cpd, and element size units such as minutes of arc (′), have an inverse relation and therefore opposite meanings of “low” and “high”. We have used “VEP SF limit” to describe the performance limit as measured by VEPs in preference to alternatives such as VEP SF threshold, VEP acuity, VEP acuity estimate or sweep VEP acuity.

## Patient populations

Visual acuity is typically measured using subjective tests such as letter charts which require the patient to have adequate cognitive and motor function and to comply with the test process. VEP SF limits are indicated in patients who cannot or will not cooperate or satisfactorily complete behavioural acuity tests or whose cooperation is suspect. VEP SF limits are useful for estimating acuity in infants and children, particularly those with motor or learning impairments which prevent reliable measurement of behavioural acuity. Typical VEP SF limits improve rapidly over the first year of life and then more slowly, reaching adult levels between 2 and 10 years of age. In the youngest typically developing infants, VEP SF limits are much better (i.e. occur at finer SF) than behavioural acuity measured with acuity card tests based on fixation preference, but the reverse is found from around 3–5 years onwards. For this reason, inferring a behavioural visual acuity from an individual infant or child’s VEP SF limit cannot use empirical calibrations established for adults.

VEP SF limits can be a good proxy for behavioural acuity in patients with media opacities, refractive errors and primarily retinal dysfunction. In patients whose primary site of dysfunction is the macula, the optic nerve or any cerebral structure, VEP SF limits may have poorer accuracy and precision when compared to behavioural measures: this includes amblyopic patients in whom VEP SF limits are relatively insensitive to reduced optotype acuity. VEP SF limits are particularly helpful in patients with non-organic vision loss providing sufficiently fine SFs are used. A VEP SF limit should be ordered and interpreted only as part of a fuller assessment and cannot be interpreted without full clinical assessment and history.

## Technical issues

**Fig. 1:**
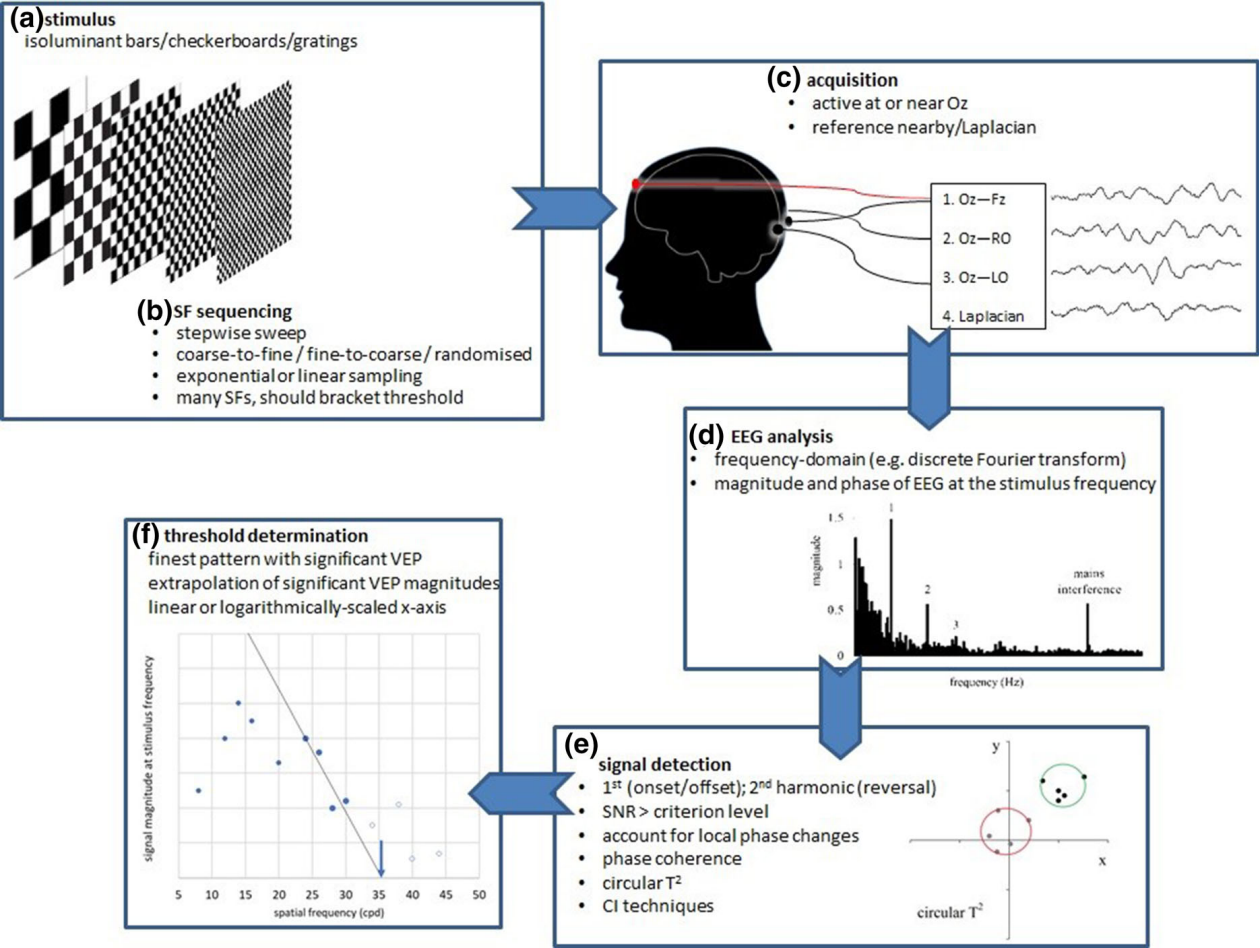
Illustrative overview of processes used to measure VEP SF limits. Panel a/b illustrates options for stimuli (gratings or checkerboards) and sequencing. Panel c illustrates a possible four-channel acquisition montage with one channel emulating the ISCEV VEP standard, Oz–Fz [1], two using closely positioned reference electrodes over the right occiput (RO) and left occiput (LO) (Oz–RO and Oz–LO) and the fourth using a Laplacian montage, Oz–((RO + LO)/2). Panel d illustrates an example of frequency domain analysis, with steady-state VEPs evident as the numbered spikes at the stimulus frequency and higher harmonics. Panel e illustrates one signal detection technique, the circular T^2^ (red circle encloses the origin and represents a non-significant steady-state VEP; green circle excludes the origin and illustrates a significant steady-state VEP)—other statistical techniques are also listed. Panel f illustrates one method for defining VEP SF limit, namely linear regression and extrapolation of the significant VEP magnitudes in the descending limb at the finest spatial frequencies: alternative methods are listed. SF, spatial frequency; SNR, signal-to-noise ratio; CI, confidence interval; cpd, cycles per degree

A broad overview of commonly used techniques is shown in Fig. 1.

- *VEP stimuli* VEP amplitude is tuned to temporal frequency and largest for stimuli which change in the range of 5–12 Hz. For reversing stimuli, there are two reversals in each cycle, so 5–12 Hz is equivalent to 10–24 reversals per s (rps). Within this approximate range, VEP SF limits are relatively constant. VEP SF limits improve with increasing mean luminance, reaching stability across the range of 25–100 cd/m^2^. Generally, higher contrast improves signal-to-noise ratio (SNR) and hence VEP SF limits, but contrast levels > 40% (Michelson) enhance a well-recognised amplitude notch (reduced amplitude VEPs at intermediate SFs) in the amplitude versus SF tuning curve, risking underestimation of VEP SF limits. Lower contrast also reduces the risk of luminance artefacts and may be more comfortable to view. VEP SF limits remain relatively stable over a large range of field sizes (2–12°); larger field sizes may compensate a little for poor fixation. Checkerboards, sinusoidal gratings and square wave gratings (bars) are widely used. While sinusoidal gratings are spatially simpler, containing a single SF, the sharp edges of square wave gratings or checkerboards contain multiple finer SFs and may provide a better accommodative stimulus. Grating orientation (horizontal vs vertical) does not affect VEP SF limits, but oblique orientations give poorer VEP SF limits, i.e. at lower SF, than cardinal orientations. A checkerboard’s fundamental SF (SF_f_) is oriented obliquely (see Eq. 1) at 45 and at 135 degrees, so VEP SF limits to checkerboard stimuli may be poorer than those to grating stimuli. Reversing stimuli produce a more marked notch in the SF tuning curve than onset/offset stimuli. Brief onsets (e.g. 40 ms) cause the on- and off-responses to overlap, producing a larger VEP than longer onsets (e.g. 300 ms [1]).
- *Stimulus sequencing* True sweep VEPs, i.e. continuously changing SFs, have been superseded by “stepwise sweep” methods, where SF is changed in discrete steps. Extrapolation techniques require adequately dense and extensive sampling of the VEP amplitude versus SF function, especially with reversing stimuli which may produce a notched function, i.e. reduced amplitude at intermediate SFs. In healthy adults and older children, patterns of up to 40 cpd may be required in order to approach or bracket their VEP SF limit and avoid underestimation errors. Linear sampling of SF produces desirably fine sampling towards the VEP SF limit of normal adults, but linear changes in SF cannot always be achieved for the finest patterns available on a display, e.g. 1 × 1 to 2 × 2 to 3 × 3 pixels. Exponential sampling gives equal weight to each octave of SF, as for a psychophysical tuning function, but spatial resolution is reduced towards the acuity limit. For sequential SF presentation, the direction of change (coarse-to-fine or fine-to-coarse) has little or no effect on VEP SF limits, although patients may be more attentive to coarse-to-fine stepwise sweeps.
- *Acquisition and analysis* Active electrodes close to Oz are optimally positioned to define VEP SF limits well. Closely positioned reference electrodes, especially in a Laplacian montage, enhance SNR towards threshold by cancelling remote noise. VEPs acquired at rates of 5–12 Hz (onset/offset) or 10–24 rps are usually analysed in the frequency domain using a discrete Fourier transform (DFT), sometimes after time-domain averaging. Epochs containing artefacts can be rejected in real time or post hoc and excluded from any time-domain average or other analysis. Typically, only the first harmonic (response at the stimulus frequency for onset/offset stimuli) or the second harmonic (response at the reversal rate for reversing stimuli) is considered since magnitude is usually lower for higher harmonics. Incorporating higher harmonics, for example by summing all harmonics which are significantly greater than noise, may be useful for improving overall SNR. The presence or absence of a VEP at the stimulus frequency is determined objectively, for example SNR ≥ 3. Noise can be estimated as the magnitude of an adjacent frequency bin in the DFT spectrum, or as the mean of the two adjacent bins, or from no-stimulus recordings. DFT phase data may be incorporated into decision-making by requiring physiologically plausible phase lead or lag with decreasing or increasing SF, respectively. Both magnitude and phase can be employed in bivariate techniques such as the circular *T*^2^ statistic or magnitude-squared coherence statistic.
- *Defining the VEP SF limit* Extrapolation technique: For each SF used as a stimulus, the VEP magnitude (μV) is plotted versus SF (cpd). Typically, this SF tuning function drops towards zero at finer SFs (see example plot, Fig. 1, panel f). Selecting only those points on this final descending portion, and performing linear regression through them, allows extrapolation of the straight line to 0 μV or to a noise “floor”: the point of intersection defines the VEP SF limit. Regression is typically performed on only significant VEP magnitudes. The SF axis may be linearly or logarithmically scaled. If extrapolation is performed to 0 μV rather than to a noise “floor”, VEP magnitudes may be adjusted for noise by subtracting a noise estimate.
  Finest SF technique: A VEP SF limit can also be defined as the finest SF evoking a significant VEP, with due regard to suitable thresholding, i.e. significant VEPs to slightly coarser SFs and absent VEPs to slightly finer SFs. This technique may produce VEP SF limits which are slightly poorer than those found by the extrapolation technique. The finest SF technique may be used as an alternative, integrated strategy when the extrapolation technique fails to define a VEP SF limit.
- *Transient VEPs and transient VEPs for SF limit measurement* ISCEV standard transient VEPs are recorded to checkwidths of 60′ and 15′ (0.71 and 2.8 cpd). It is not advisable to attribute an acuity based on their presence, absence or normality. For example, an extant VEP (transient or steady-state) to a 15′ (2.8 cpd) checkwidth pattern would be in keeping with “good visual acuity” for a baby, but as a limit, this would be much poorer than typical for any patient aged over 1 year. In some cases, the presence of a transient VEP may suggest grossly better visual acuity than reported subjectively, for example in cases of severe non-organic visual loss, but this observation lacks precision regarding a SF limit.
  Transient VEP SF limits can be used to estimate acuity, but it takes much longer than steady-state VEP methods to evoke reproducible responses to multiple SFs, and limits are therefore more prone to be affected by patient fatigue and neural adaptation. Furthermore, objective detection techniques for transient VEPs have not been widely adopted and subjective recognisability of transient VEP waveforms risks inter-operator differences in limits; transient VEPs are therefore not included this extended protocol.

## Calibration

Calibration of stimulation and recording systems should be verified and re-calibrated if indicated at intervals as specified in the current ISCEV VEP standard and calibration guideline [1, 5]. It is particularly important that users ensure the absence of any luminance artefact such as transient artefacts created by non-CRT screens or artefacts introduced in onset/offset stimuli due to luminance or spectral differences between the grey background and the pattern. All pattern element sizes, for example checkwidths, should be directly measured to verify the visual angle subtended. Patterns should be expressed in cpd using appropriate conversion formulae (Table 1 in [2]). In particular, the obliquely oriented SF_f_ (cpd) of a checkerboard is expressed as

1 $${\text{SF}}_{\text{f}} = \frac{60}{{\sqrt 2 w_{\text{c}} }}$$

where *w*_c_ is the visual angle subtended by one checkwidth in minutes of arc.

An empirical calibration factor is required to infer a behavioural acuity from a VEP SF limit: see Response evaluation part (c) below.

## Protocol specifications

Patient preparation follows that of the current VEP standard [1], except for the reference electrode placement (see (e) Electrode montage, below). Measurement of the VEP SF limit may precede or follow ISCEV standard minimum protocols. Binocular stimulation is used when the aim is to gain insight into practical functioning levels. Monocular testing is used when interocular differences are suspected. Patients should be physically well supported which may mean using a carer’s lap with heads supported securely for infants or small children or the patient’s own mobility chair.

- *Fixation and ambient lighting* Fixation should be closely monitored during recording and acquisition suspended during poor fixation. The quality of the EEG signal should be monitored, and automated artefact rejection can be used. Ambient lighting should be chosen to maximise the patient’s attention and fixation on the stimulus screen.
- *Refraction and mydriasis* Patients should wear any required refraction, and pupils should not be pharmaceutically dilated. Cycloplegia and optimal refractive correction for the fixation distance may be useful for some patients, for example in some cases of suspected malingering or factitious disorders.
- *Spatial and temporal stimulus parameters* This protocol requires vertical or horizontal sinusoidal or square wave gratings, or checkerboards, presented in either onset/offset or reversal mode with a temporal frequency of 5–12 Hz (onset/offsets) or 10–24 reversal per s (reversals), to evoke steady-state VEPs. Onsets should be brief, for example 40 ms, and should not exceed 60 ms. Mean luminance should be approximately 50 cd/m^2^ (acceptable range 25–100 cd/m^2^). Michelson contrast around 40% is specified to minimise any notch in the SF tuning curve and commensurate risk of VEP SF limit underestimation: however, higher contrasts may be used if needed for better SNR, for example in pathologies affecting contrast sensitivity. A field size > 15°, as for the VEP standard, is suitable, but may need to be smaller to accommodate increased viewing distances required for fine SFs: field size should not be less than 3° in diameter. Field sizes larger than 15° may help compensate for poor fixation or to enable display of the coarsest SFs.
- *Sequence and range of stimuli* The range of SFs should be tailored to the needs of each patient as far as possible, with the finest SFs presented being beyond their VEP SF limit. Either linear or exponential (i.e. linear on a logarithmically scaled axis) sampling of SF is suitable. Successful strategies tend to use 8–20 SFs, although fewer are possible if there are sufficient points above and below the VEP SF limit. Coarse-to-fine or fine-to-coarse SF sequencing is acceptable, as is random, pseudo-random or staircasing sequences. Patients may be more attentive to coarse-to-fine stepwise sweeps.
- *Electrode montage* A single channel recording with the active electrode at Oz, as for ISCEV standard VEPs, is adequate; the reference electrode is closely positioned, for example at O1, O2 or Pz. Two or more channels, for example Oz referenced to O1 and Oz referenced to O2, may be used, and data from whichever channel has the highest SNR can be selected. Channels could use montages with more distant reference electrodes, e.g. Cz or Fz, as for ISCEV standard VEPs. Using a Laplacian montage to enhance VEP detection is also acceptable and can be implemented using two close reference electrodes, e.g. O1 and O2, and a “virtual” channel derived as Oz − ((O1 + O2)/2). A separate electrode, at a site such as the forehead, vertex (Cz), mastoid or earlobe, should be connected to the ground.

## Response evaluation

- *Data analysis* Magnitude and phase of the EEG signal at the stimulus frequency for onset/offset or at the reversal rate for pattern-reversal stimuli should be determined with a suitable technique, for example the DFT. Artefact rejection should be used, e.g. exclusion of epochs containing artefact from time-domain averaged data. Significance of the signal should be established objectively based on magnitude and/or phase statistics.
- *VEP SF limit* The VEP SF limit is defined as the extrapolated limit or the finest SF evoking a significant VEP. Both magnitude and phase plots with axes labelled with relevant units (e.g. μV and degrees vs stimulus SF_f_ (cpd)) should be inspected for physiologically plausible findings, e.g. reasonable magnitudes and phase lag increasing with SF. Plots should indicate which SFs evoked significant or non-significant VEPs and which were used for any regression. To meet this extended protocol, all users should pre-specify rules for determining the limit. These rules may be automatically applied or may have a manual element, such as selecting points for regression or excluding artefacts that meet specific criteria. Any manual rules should be pre-specified and applied identically by all operators to avoid different operators finding different limits from the same data. VEP SF limits at this stage should be described as the VEP stimulus at the SF limit (cpd) and not converted into an estimate of behavioural acuity.
- *Option of inferring a behavioural acuity* If VEP SF limits are used to infer a behavioural acuity, an empirical calibration factor is required. This should be derived empirically from an adequately sized group of subjects from whom both VEP SF limits and behavioural acuities have been obtained. Some measure of the spread of values, for example limits of agreement, as well as a point estimate of the average offset should be given. Empirical calibration factors derived from adult subjects are not valid for infants or children younger than 3–5 years old. Where a calibration factor from elsewhere is used, e.g. from independent studies or as part of a manufacturer’s protocol, its provenance should be available in sufficient detail to allow new users to judge its transferability to their patient population.

## Reporting

Full details of all stimulus, acquisition and analysis parameters, pertinent recording details such as quality of fixation and plots of VEP magnitude and phase vs SF should be included or available. The plots should indicate all SFs which were employed and those which evoked significant VEPs. If extrapolation is used, the regression line and the SFs regressed should also be indicated. If extrapolation is not used, the criterion for the VEP SF limit should be stated, i.e. the finest SF evoking a significant VEP. Limits should be given in cpd. Reports should state age-appropriate reference intervals in cpd, including their provenance, and a statement of normality or otherwise for the patient tested.

There is no requirement for the further step of relating the VEP SF limit to behavioural acuity measures. If this is undertaken, reports should explicitly state what empirical calibration factor has been applied with access to a reference for its provenance which provides details such as ages of subjects, behavioural acuity tests used and a measure of variability, for example limits of agreement. Reports should advise caution with interpreting results for patients whose known or suspected type of visual dysfunction potentially makes their VEP SF limit an unreliable estimate of behavioural acuity.

## Appendix: Justification of the protocol details

The committee was formed of individuals from diverse centres with experience of development and/or use of clinical VEP SF limits. To minimise bias, a systematic review was undertaken. In brief, four databases were independently searched using appropriate MeSH terms or equivalent keywords for studies (articles, conference proceedings or dissertations) describing VEPs used to estimate visual acuity in humans. Titles and abstracts and, where necessary, full texts were screened to identify potentially eligible studies for inclusion. Data were extracted from included studies using a standardised template. The protocol was registered with the international prospective register of systematic reviews (PROSPERO), registration number CRD42018085666, and methodology is reported according to the Preferred Reporting Items for Systematic Reviews and Meta-Analyses (PRISMA) statement [6]. This extended protocol is an informed distillation of the findings of the systematic review [2].
